# Bidirectional association of neurodevelopment with growth: a prospective cohort study

**DOI:** 10.1186/s12887-021-02655-7

**Published:** 2021-04-28

**Authors:** Xiaotong Wei, Jiajin Hu, Liu Yang, Ming Gao, Lin Li, Ning Ding, Yanan Ma, Deliang Wen

**Affiliations:** 1 Institute of Health Sciences, China Medical University, No.77 Puhe Road, Shenyang North New Area, Shenyang, 110122 Liaoning P.R. China; 2 Department of Obstetrics and Gynecology, Shenyang Women and Children Health Care Centre, Shenyang, Liaoning China; 3 Department of Developmental Pediatrics, Shengjing Hospital of China Medical University, Shenyang, Liaoning China; 4 Curriculum and Teaching Research Office, Research Center of Medical Education, China Medical University, Shenyang, Liaoning China; 5 Department of Epidemiology and Health Statistics, School of Public Health, China Medical University, Shenyang, 110122 Liaoning China

**Keywords:** Neurodevelopment, Anthropometric measurements, Pediatrics, Physical growth

## Abstract

**Background:**

The study aims to use the cross-lagged model and utilize data from the Born in Shenyang Cohort Study to characterize the bidirectional associations of the term-born infants’ neurodevelopment in five domains and physical growth in early life.

**Method:**

This study consists of 688 mother-child dyads from the Born in Shenyang Cohort Study. Infants’ anthropometric (weight and length) and development in neurological outcomes (Gesell Development Scale) were measured at the age of 6 and 12 months. Cross-lagged analyses and multiple linear regression analyses were used to explore the longitudinal relationships in both directions.

**Results:**

In terms of longitudinal studies, the inverse associations between infants’ two skills (gross motor and social behavior) at the age of 6 months with BMI Z -scores at the age of 12 months (gross motor: aβ = − 0.20, 95% CI: − 0.31 to- 0.09; social behavior: aβ = − 0.23, 95% CI: − 0.33 to- 0.13) were found. Conversely, a higher infant Z -scored BMI at the age of 6 months predicted a lower gross motor at the age of 12 months (aβ = − 0.08, 95% CI: − 0.12 to- 0.04). In cross-lagged analyses, an adverse association in both directions between gross motor and Z -scored BMI was observed.

**Conclusion:**

We found bidirectional relationships between infants’ neurodevelopment of gross motor with physical growth and suggested the term-born infants, who are on the edge of the developmental danger, should not be overlooked.

**Supplementary Information:**

The online version contains supplementary material available at 10.1186/s12887-021-02655-7.

## Background

The relationships between physical growth and neurodevelopment are found; however, the literature for infants, especially within term-born, is relatively few [1, 2]. As far as we know, the outstanding cohort study, which named Avon Longitudinal Study of Parents and Children (ALSPAC), has paid attention to this relationship. They found that physical growth in adults and children were not associated with neurodevelopment, suggesting that physical development in early life may be a critical period of later neural development [3]. Between birth and 1 year, body weight has triples, body length increases by more than 50%, and brain volumes increase to 75% of an adult’s size [4].

Besides, although recent studies have found that bidirectional effects between physical growth and neurodevelopment, that is, physical growth may be both influencing and responding to infants, few studies have the analysis of these relationships. On the one hand, physical growth may be a sign of disruption of the critical steps during brain development. The brain and nervous system growing at its fastest rate during this time, such as weight status and weight gain during this process, can have long-term impacts on the brain’s developing structure and function [5–8]. Moreover, research addressing children’s physical growth on neurodevelopment has shown controversial findings. Some studies found that weight status has been negatively associated with neurodevelopment, especially in motor development and cognitive development. However, longitudinal studies confirmed no temporal associations in the population [9–12]. On the other hand, the state of neurodevelopment in young children might also affect physical development [13, 14]. However, few epidemiologic studies that focus on the effects and have shown controversial findings [15]. Although a comprehensive systematic review study showed infants with better development in psychiatric and neurological demonstrated a greater gain in height and weight gain between 4 and 12 months, the study to explore early development (SEED) showed infants with intellectual disability and neurodevelopmental impairments, in particular, would be at greater risk for rapid weight gain or obesity during infancy [16].

The bidirectional relationships between early life neurodevelopment with physical growth can be affected by numerous confounding factors. As these factors are often complex and interdependent, using independent regression models is cumbersome. In this paper, the study needs to explore the relationships between physical growth and neurodevelopment during infancy, and investigate causal pathways beginning in early life. The cross-lagged analysis should be used to overcome these problems, which is a powerful statistical approach to address methodological problems.

In this study, we considered to use the cross-lagged models and utilize data from the Born in Shenyang Cohort Study (BISCS) to characterize the bidirectional associations of term-born infants’ neurodevelopment in five domains (adaptive behavior, gross motor, fine motor, language, and social behavior) and physical growth (Body Mass Index (BMI) *Z* -score and weight gain velocity) in the early life.

## Methods

### Population and study design

**Fig. 1:**
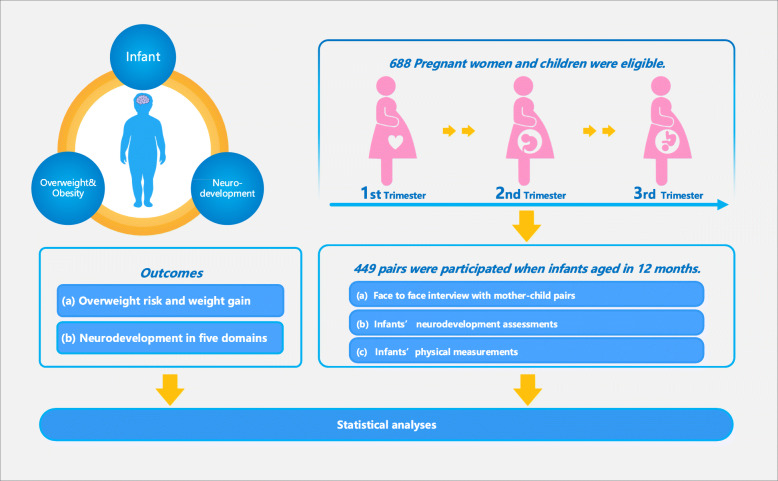
Flowchart of recruitment and research

The BISCS was a representative, prospective cohort study conducted by China Medical University between 2017 and 2020. We enrolled pregnant women from 54 hospitals and community health care centers, which were all perinatal care institutes that provide antenatal and maternity care in the urban areas of Shenyang, located in northeast China [17]. In-person visits were conducted with mothers between 13 and 27 weeks and 28–36 weeks of gestation to record maternal demographic information and collect biological samples. The visits were conducted again at the age of 1, 3, 6 and 12 months postpartum to record maternal postnatal data and infant information, and use the Gesell Developmental Scale to assess the infant neurodevelopmental levels at the age of 6 and 12 months. Children born to mothers before 36 weeks gestation, with severe complications during pregnancy, diseases in the nervous system around birth, as part of a multiple pregnancies (e.g., twins, triplets), or without complete baseline and follow-up data were excluded. As Fig. 1, a final cohort of 688 women was completed at delivery, at the age of 6 months postpartum (*n* = 459), and at the age of 12 months postpartum (*n* = 449). There was no difference found in maternal and infant demographic information between attrite participants and those with complete information (see Table S1).

### Measures

#### Anthropometric outcomes

The weight and length at birth of the infants were recorded from the medical records, and at the age of 6 months (mean: 6.87 ± 1.27) and at the age of 12 months postpartum (mean: 11.67 ± 1.41) by BISCS staff, according to a standard protocol. The weight and length were measured with a digital scale and a stadiometer while children were wearing no shoes and light clothes (Seca 416 and 376 +; Seca Corporation, Hamburg, Germany). Birth *Z* -score, *Z*-scored BMI for age, sex-specific weight for age (WFA) and weight-for-length (WFL) were calculated based on the World Health Organization (WHO) child growth reference [18]. Weight gain velocity was calculated as the change in WFA *Z* -score from birth to 6 months and 6 months to 12 months [19].

#### Infants’ neurodevelopment

Participants in this birth cohort were administered the Gesell Development Scale (GDS), which has been used extensively after being translated and standardized [20]. The Gesell Development Scale is used to evaluate the function of the central nervous system and identify defects in the neuromuscular or sensory system, which consists of five domains, including the adaptive domain (coordination, imitation, object recovery, discrimination and perception), gross motor domain (changes in posture, head balance and behavioral observations of standing, sitting and walking) and fine motor domain (ability to hold objects with fingers), language domain (vocabulary comprehension and dialogue skills), and social domain (social habits, reactions to persons, autonomy and independence). Developmental quotient (DQ) is defined as the quotient between developmental age and actual age, and the DQ of each child was calculated for each specific domain. Higher developmental quotient (DQ) scores mean higher cognitive levels [21]. Two well-trained pediatricians from the Shengjing Hospital of China Medical University assessed all participants to maximize reliability. They assessed all children in their assigned domain to avoid interexaminer variability.

#### Covariates

Based on previous research [22–25], other variables were used to describe maternal and infants’ demographic characteristics and control for confounding variables. These variables were collected from hospital records (maternal age, maternal pre-pregnancy BMI [26], gestational weight gain (GWG), delivery mode, parity, child sex, gestational weeks, and birth weight) and questionnaires (maternal education, annual family income, primary caregiver, mode of infant feeding, the introduction of solid foods at the age of 6 months and micronutrients supplementation (Iron, Vitamin B12 and Zinc) at the age of 6 months). Only 0.85% of women smoked and 1.15% of women were drinking during pregnancy, so this variable was not included. We calculated the GWG rate (kg/week) as the total GWG divided by the number of gestational weeks at delivery. Based on the IOM, above optimal weight gains are above 16 kg for mothers with normal weight and more than 11.5 kg for overweight mothers, respectively [27, 28]. We categorized the mode of infant feeding as: formula feeding only, mixed breast and formula, and breast milk feeding only. Micronutrient supplementation (Iron, Vitamin B12 and Zinc) was treated as a dichotomized variable (yes/no). To ensure data integrity, the wholly conditional specification multiple imputation (m = 20) was used to impute missing data.

### Statistical analyses

#### Longitudinal analyses

Multiple linear regression analyses were fitted to assess the association of infants’ BMI *Z* -score and weight gain velocity with neurodevelopmental status at the age of 6 and 12 months. The analyses used repeated measured weight status and neurodevelopmental variables at two-time points. The analyses were adjusted for all the above confounding factors. Furthermore, the associations between neurodevelopmental status at the age of 6 months and weight status at the age of 12 months were additionally adjusted for weight status at baseline (6 months) to study whether neurodevelopmental status predicted the change in infants’ *Z* -scored BMI or weight gain velocity, and vice versa. The above analyses were also carried out in the relationships between Z -scored WFL and neurodevelopmental status. Linear regression analyses were run using SPSS version 20.0 (SPSS 20.0, [2011], IBM).

#### Cross-lagged analyses

This cross-lagged analysis included confounders, stability effects, cross-sectional associations, longitudinal associations, and cross-lagged associations. Firstly, we examined the stability model, which only included the cross-sectional analysis, with confounding factors regressed at two baselines. Then, based on the first step, we entered the lagged association between infant BMI *Z* -score and weight gain velocity at the age of 6 months with neurodevelopmental status at the age of 12 months, and the lagged association between neurodevelopmental status at the age of 6 months with infant BMI *Z* -score and weight gain velocity at the age of 12 months. Finally, the above two lag associations entered the whole model at the same time. The above relationships were adjusted for all the above confounding factors. Besides, the cross-lagged associations were adjusted for weight status at baseline (the age of 6 months) or neurodevelopmental status at baseline (the age of 6 months). The cross-lagged analyses were conducted with Mplus, version 7.11.

## Results

### Participants characteristics

Characteristics at follow-up at the age of 6 and 12 months were shown (Table S1, see Supplemental materials). Among eligible mother-offspring pairs, the mean (SD) age of the recruited mothers was 30.4 ± 3.9 years, and about a quarter of mothers were overweight. Four hundred and fifty-nine infants (234 [51.1%] were boys, 225 [48.9%]) were girls who assessed at the age of 6 months. At the age of 6 and 12 months, the mean infants’ BMI Z- score were 0.16 ± 0.97 and 0.23 ± 1.05, respectively. Nearly one-fourth of infants went through rapid weight gain, and at the age of 6 months and at the age of 12 months, 47 (10.2%) and 51 (11.4%) were overweight, respectively. The infant average DQ scores in domains of adaptive behavior, gross motor, fine motor, language, and social behavior were 97.9, 100.3, 94.1, 85.9, and 93.2 at the age of 6 months, respectively. Furthermore, the mean scores were 103.3, 101.2, 104.8, 94.9, and 112.7 at the age of 12 months, respectively.

### Longitudinal analyses

As shown in Tables 1 and S2 (see Supplemental materials), the linear regression analysis evaluated the associations between neurodevelopmental status at the age of 6 months and *Z* -scored BMI at the age of 12 months. Results found that an additional unit of infants’ two skills (gross motor and social behavior) at the age of 6 months could be decompensated by 0.2 SD decrease (0.23 SD decrease) in BMI at the age of 12 months (gross motor: aβ = − 0.20, 95% CI: − 0.31 to- 0.09, *P* = 0.002; social behavior: aβ = − 0.23, 95% CI: − 0.33 to- 0.13, *P* = 0.005). No effect of the other three neurodevelopment skills was observed. Similar associations had been found between the DQ scores at the age of 6 months and the risk of rapid weight gain or Z -scored WFL at the age of 12 months in Tables S2 and S4 (see Supplemental materials).

**Table 1:** Longitudinal associations between neurodevelopment and BMI z-scores at 6 and 12 months of age^a^

Outcomes at 12 months	Predictors at 6 months		β (95% CI)
Infant Z-scored BMI	Adaptive behavior	Model 1	−0.06(− 0.20,0.08)
		Model 2	−0.04(− 0.18,0.10)
		Model 3	−0.03(− 0.19,0.13)
	Gross motor	Model 1	−0.54(− 0.67,-0.41)*
		Model 2	−0.44(− 0.58,-0.30)*
		Model 3	−0.20(− 0.31,-0.09)*
	Fine motor	Model 1	−0.03(− 0.15,0.09)
		Model 2	−0.03(− 0.16,0.11)
		Model 3	−0.01(− 0.05,0.04)
	Language	Model 1	−0.03(− 0.13,0.07)
		Model 2	−0.03(− 0.12,0.07)
		Model 3	−0.02(− 0.10,0.06)
	Social behavior	Model 1	−0.35(− 0.48,-0.23)*
		Model 2	−0.31(− 0.41,-0.21)*
		Model 3	−0.23(− 0.33,-0.13)*

Model 1: adjusted for basic information; Model 2: model 1+ maternal pre-pregnancy BMI, gestational weight gain, delivery mode, gestational weeks, birth weight z score, mode of infant feeding, introduction of solid foods and micronutrients supplementation; Model 3: model 2+ Infant Z-scored BMI at 6 months in the neurodevelopment- BMI relationships, or neurodevelopment scores at 6 months in the BMI – neurodevelopment relationships

*Statistically significant

^a^*N* = 449. N varied from 1.7 to 2.8% in each regression because the complete data for each subscale of the Gesell Development Scale were varied

As shown in Table 2, the linear regression analysis assessed the associations between *Z* -scored BMI at the age of 6 months and neurodevelopmental status at the age of 12 months (Table 2). Each SD increase in infant Z -scored BMI at the age of 6 months was associated with 0.08 unit reduction in the gross motor domain at the age of 12 months (aβ = − 0.08, 95% CI: − 0.12 to- 0.04, *P* = 0.03). However, *Z* -scored BMI at the age of 6 months showed a negative correlation with social behavior skills at the age of 12 months, which tends to disappear when considering social behavior skills at the age of 6 months as a confounding variable (model 2: aβ = − 0.08, 95% CI: − 0.11 to- 0.05, *P* = 0.03; model 3: aβ = − 0.05, 95% CI: − 0.08 to 0.00, *P* = 0.06). Like *Z*-scored BMI results, the relationships between weight gain velocity or Z -scored WFL and at the age of 6 months with neurodevelopmental status at the age of 12 months were performed in Tables S3 and S5 (see Supplemental materials).

**Table 2:** Longitudinal associations between BMI z-scores and neurodevelopment at 6 and 12 months of age^a^

Outcomes at 12 months	Predictors at 6 months		β (95% CI)
Adaptive behavior	Infant Z-scored BMI	Model 1	−0.01(− 0.03,0.01)
		Model 2	−0.01(− 0.02,0.01)
		Model 3	−0.01(− 0.02,0.01)
Gross motor		Model 1	−0.29(− 0.36,-0.22)*
		Model 2	−0.19(− 0.29,-0.09)*
		Model 3	−0.08(− 0.12,− 0.04)*
Fine motor		Model 1	-0.04(−0.11,0.03)
		Model 2	−0.02(− 0.06,0.03)
		Model 3	−0.02(− 0.04,0.01)
Language		Model 1	−0.02(− 0.04,0.01)
		Model 2	−0.02(− 0.04,0.01)
		Model 3	−0.02(− 0.03,0.01)
Social behavior		Model 1	−0.09(− 0.12,− 0.08)*
		Model 2	-0.08(−0.11,− 0.05)*
		Model 3	-0.05(−0.08,0.00)

Model 1: adjusted for basic information; Model 2: model 1+ maternal pre-pregnancy BMI, gestational weight gain, delivery mode, gestational weeks, birth weight z score, mode of infant feeding, introduction of solid foods and micronutrients supplementation; Model 3: model 2+ Infant Z-scored BMI at 6 months in the neurodevelopment- BMI relationships, or neurodevelopment scores at 6 months in the BMI – neurodevelopment relationships

*Statistically significant

^a^*N* = 449. N varied from 1.7 to 2.8% in each regression because the complete data for each subscale of the Gesell Development Scale were varied

### Cross-lagged analyses

**Fig. 2:**
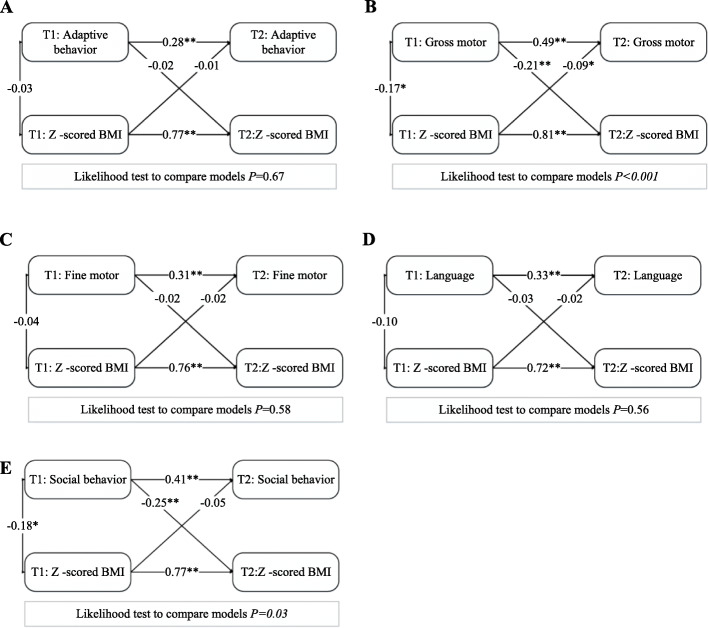
Cross-lagged model of associations between neurodevelopment and z-scored BMI at the age of 6 and 12 months (*N* = 449). The values represent β-regression coefficients and adjusted for confounding variables. The model fit well among the five models, and ranged from: Χ^2^ = 6.86–20.98, RMSEA = 0.02–0.05, CFI = 0.96–1.00, TLI = 0.98–1.00, SRMR = 0–0.02. T1:6 months; T2: 12 months; BMI: body mass index. *P < 0.05, **P < 0.001

**Fig. 3:**
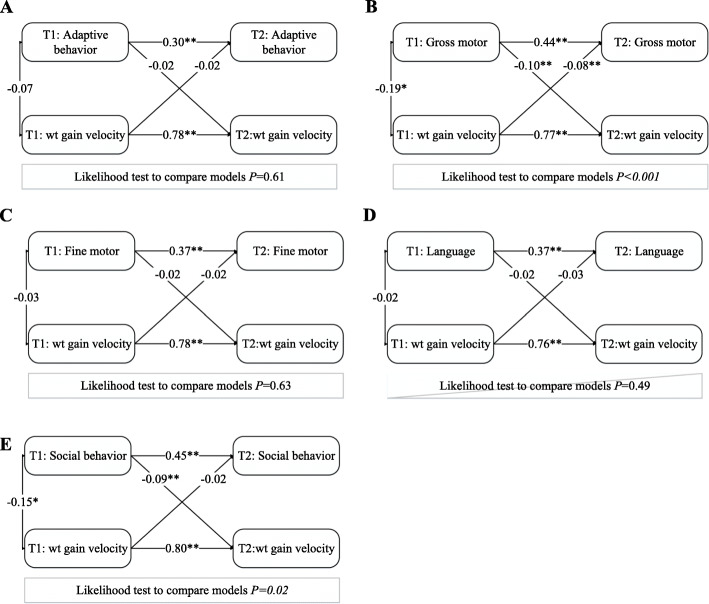
Cross-lagged model of associations between neurodevelopment and weight gain at the age of 6 and 12 months (*N* = 449). The values represent β-regression coefficients and adjusted for confounding variables. The model fit well among the five models, and ranged from: Χ^2^ = 8.86–27.98, RMSEA = 0.01–0.04, CFI = 0.97–1.00, TLI = 0.95–1.00, SRMR = 0.00–0.02. T1:6 months; T2: 12 months; wt: weight. *P < 0.05, **P < 0.001

The cross-lagged models of bidirectional associations between infant neurological development levels and *Z* -scored BMI at 6 and 12 months were shown in Fig. 2. All cross-lagged analyses demonstrated SRMR and RMSEA were close to zero, and TLI and CFI were above 0.9, which means the degrees of fitting were good. An adverse association in both directions between gross motor and *Z* -scored BMI was observed. The negative relationship of social behavior at the age of 6 months of age with BMI *Z* -score at the age of 12 months was found, but there was not differed statistically in the opposite direction. Similar to *Z*-scored BMI results, of bidirectional associations between weight gain velocity with neurodevelopmental status were performed in Fig. 3.

## Discussion

To the best of our knowledge, the current study is one of the few to explore the bidirectional associations between neurodevelopment, as assessed by the Gesell Development Scale, and physical growth among term-born infants. Our birth cohort study suggests the negative associations between gross motor and social behavior at the age of 6 months with weight status at the age of 12 months. Regarding the opposite direction of relationship, a higher infant *Z* -scored BMI at the age of 6 months predicted a lower neurological development level in the gross motor at the age of 12 months. Gross motor performed a bidirectional relationship, although neurological development level had a more significant effect on infants’ BMI *Z* -score than the opposite. Similar associations had been found in weight gain velocity.

In motor abilities, our findings are consistent with previous studies demonstrating that gross motor skills were negatively associated with infant physical growth in both directions [29]. Our results indicate a bidirectional causal temporal relationship between gross motor skills and infant physical growth, which means that an increase in physical index or in gross motor capacity can cause each other to decline. In clinical practice, more attention should be paid to physical development or neural development, which is beneficial to strengthen the predictive function of each other. However, Schmidt et al. found minimal correlations between motor development and weight status, suggesting that weight status and motor milestones are mostly independent of one another [30]. There are several possible reasons for these differences. First, the samples were analyzed in different age groups. Most recent studies concentrate on exploring relationships in preschool children or school-aged children, whereas the participants in our study were infants, especially within term-born infants. Second, differences may have arisen due to the fact that different tools measured the participants. The current study evaluated motor skills using the GDS, which were in line with motor milestones and dependent on the age interval of the infants. However, this scale is still heterogeneous with other measurement tools. Potential mechanisms for these effects include the possibility that infants with poor gross motor performance are frequently associated with biomechanical problems and morphological constraints on tasks involving changes in overweight/obese status and fat mass [31]. However, Gentier and colleagues suggested that a deficit in childhood motor skills should be examined in a broader sense, rather than a mechanical interpretation. For example, it is plausible that the reduced physical activity and the lack of decision-making, planning, and control functions in infants with poor motor ability are causal factors in obese status, suggesting that impaired motor development is also related to children with obesity [12]. Therefore, additional exploratory analyses to determine the bidirectional associations between motor domains and weight status should be conducted.

Consistent with our results, previous studies observed the relationships between social skills and infant’s physical development [32]. According to the Infant Research framework [33], infant social communication abilities develop in dynamic functional interactions between mothers and infants. Ostensibly, it should be recognized that the relationship between mother and infant is bidirectional and may influence infant clues’ accuracy. As infants grow, they use communicate abilities to express wants or dislike certain foods [34]. Suppose infants have poor social skills, such as infrequent laughter, reduced focus, shared attention, and/or unclear signals to articulate their needs (including the cues of full or hungry). In that case, it may preclude their potential development of caregivers’ feeding responsiveness [35]. Scholars propose that maternal responsiveness to their children’s expressions, such as appetite, hunger, and satiety is essential when developing a healthy diet and could play an essential role in offspring weight status [32]. Besides, it is generally known that language and social competence can be simultaneously discussed as previous studies have demonstrated that children’s social and language development are synchronized mainly [36]; however, no relationship between language ability and body weight was found in our study. Additional research is needed to investigate further the potential contribution of children’s social communication skills and language ability to maternal factors.

Our prospective study provided a unique opportunity to explore the bidirectional relationships between infants’ neurodevelopment and physical growth and had attempted to analyze the associations of mutual prediction, which have been of little attention to date. In our study, the bidirectional relationships between term-born infants’ neurodevelopment of gross motor with physical growth are found. However, there are also several limitations. First, our study is limited in sample size and follow-up time, then might weakly predict later attainment. Nevertheless, physical and neurological development in early life is irreversible ^3^. Based on this study, long-term follow-up data will be used to verify our research results further. Second, Confounding factors, such as feeding style (responding style, authoritative feeding, etc.), breastfeeding (bottle or exclusive breastfeeding) were not fully adjusted.

## Conclusion

Our works suggest that the development within term-born infants should not be overlooked, but only concerned children with developmental disabilities are incomplete. Strengthen knowledge education for caregivers and improve the physical examination system for infants and young children could avoid becoming a hidden danger in developmental delays.

## Supplementary Information

**Additional file 1: Supplemental Table 1**. Demographic characteristics of mother-child pairs, comparing those with and without infant BMI data

**Additional file 2: Supplemental Table 2**. Longitudinal associations between neurodevelopment and weight gain velocity at 6 and 12 months of age^1^

**Additional file 3: Supplemental Table 3**. Longitudinal associations between weight gain velocity and neurodevelopment at 6 and 12 months of age^1^

**Additional file 4: Supplemental Table 4**. Longitudinal associations between neurodevelopment and WFL z-scores at 6 and 12 months of age^1^

**Additional file 5: Supplemental Table 5**. Longitudinal associations between WFL z-scores and neurodevelopment at 6 and 12 months of age^1^

## Data Availability

The datasets supporting the conclusions of this article are included within the article. The underlying datasets are available from the corresponding author on reasonable request.

## Abbreviations

ALSPAC: Avon Longitudinal Study of Parents and Children
SEED: The study to explore early development
BISCS: Born in Shenyang Cohort Study
BMI: Body Mass Index
WFA: Weight for age
WHO: World Health Organization
GDS: Gesell Development Scale
DQ: Developmental quotient
GWG: Gestational weight gain
Wt: Weight
