# Supplementary material for: Bidirectional association of neurodevelopment with growth: a prospective cohort study
Source: BMC Pediatr. 2021 Apr 28;21:203. doi: 10.1186/s12887-021-02655-7 (PMC8080371; doi:10.1186/s12887-021-02655-7)
Supplement: Supplementary file 1 — Additional file 1: Supplemental Table 1. Demographic characteristics of mother-child pairs, comparing those with and without infant BMI data [file 12887_2021_2655_MOESM1_ESM.doc]

**Supplemental Table 1** Demographic characteristics of mother-child pairs, comparing those with and without infant BMI data.

|  | With BMI Data(*N*=459) | | Without BMI Data (*N*=229) | | *P* |
| --- | --- | --- | --- | --- | --- |
| Characteristics | *N* or *n* | Mean±SD or n (%) | *N* or *n* | Mean±SD or n (%) |  |
| Maternal, *N*, mean±SD |  |  |  |  |  |
| Age (years) | 459 | 30.44±3.94 | 229 | 31.41±4,60 | 0.78 |
| Gestational weeks(weeks) | 459 | 38.86±2.40 | 229 | 37.21±3.33 | 0.14 |
| Prepregnancy BMI, kg/m^2^ | 450 | 21.59±3.57 | 238 | 22.07±2.88 | 0.21 |
| Excessive total GWG | 450 | 113(25.11) | 238 | 64(26.73) | 0.25 |
| Infant, *N*, mean±SD |  |  |  |  |  |
| Male | 459 | 234(51.11) | 229 | 113(49.54) | 0.53 |
| Birth weight(kg)  WFA at birth, z score  WFA at 6 months, z score  WFA at 12 months, z score  Wt gain velocity, birth to 6 months,Change in WFA z score  Wt gain velocity, 6 to 12 months,Change in WFA z score  BMI at 6 months, z score  BMI at 12 months, z score | 451  451  459  449  459  449  459  449 | 3.35±0.58  0.41±0.77  0.29±0.90  0.34±0.94  -0.12±1.07  -0.19±1.21  0.16±0.97  0.23±1.05 | 237  237  -  -  -  -  -  - | 3.17±1.34  0.36±0.96  -  -  -  -  -  - | 0.34  0.57  -  -  -  -  -  - |
| WFL at 6 months, z score | 459 | 0.45±0.93 | - | - | - |
| WFL at 12 months, z score | 449 | 0.59±1.51 | - | - | - |
| Delivery mode  Cesarean Delivery  Vaginal Delivery | 459 | 285(62.22)  174(37.78) | 229 | 138(60.37)  91(39.73) | 0.44 |
| Type of infant feeding at 6 months  Formula feeding only  Mixed breast milk and formula  Breast milk feeding only | 442 | 11(2.21)  291(66.74)  140(31.05) | 246 | 10(4.06)  183(73.90)  53(22.04) | 0.07 |
| Introduction to solid foods, month | 442 | 5.11±1.53 | 246 | 4.57±0.68 | 0.12 |
| Micronutrients supplementation | 442 | 385(87.10) | 246 | 205(83.33) | 0.63 |
| Maternal education  Lower than senior high school  College or university  Higher than undergraduate | 459 | 55(11.97)  318(69.84)  86(18.19) | 229 | 37(16.34)  153(66.81)  38(16.85) | 0.33 |
| Family yearly income (RMB/yearly)  <70000  ≥70000 | 459 | 188(41.02)  271(58.98) | 229 | 103(45.18)  126(54.82) | 0.47 |
| The primary caregiver  Mother  Father  Grandparent  Others(baby sitters) | 459 | 243(52.99)  38(7.98)  187(40.80)  23(4.43) | 229 | 124(54.13)  19(8.32)  97(42.75)  11(5.10) | 0.52 |
| Parity  0 previous births  ≥1 previous births | 453 | 295(64.97)  158(35.03) | 235 | 144(61.53)  91(38.47) | 0.19 |

WFA, weight for age ; Wt, weight; BMI, Body Mass Index; WFL, weight-for-length ;GWG, Gestational weight gain.

**Bidirectional association of** **neurodevelopment with growth: A Prospective Cohort Study**

**European Journal of Pediatrics**

**Xiaotong Wei^1^, Jiajin Hu^1^, Liu Yang ^2^, Ming Gao^1^, Lin Li^3^, Ning Ding^4^, Yanan Ma^5^ and Deliang Wen^1^,***

* **Correspondence:**

Deliang Wen;

Institute of Health Sciences ,China Medical University, No.77 Puhe Road, Shenyang North New Area, Shenyang, Liaoning Province, 110122, P.R. China (email: dlwen@cmu.edu.cn), (phone: +86 024-31939003).
