# Supplementary material for: Bidirectional association of neurodevelopment with growth: a prospective cohort study
Source: BMC Pediatr. 2021 Apr 28;21:203. doi: 10.1186/s12887-021-02655-7 (PMC8080371; doi:10.1186/s12887-021-02655-7)
Supplement: Supplementary file 2 — Additional file 2: Supplemental Table 2. Longitudinal associations between neurodevelopment and weight gain velocity at 6 and 12 months of age1 [file 12887_2021_2655_MOESM2_ESM.docx]

**Supplemental Table 2** Longitudinal associations between neurodevelopment and weight gain velocity at 6 and 12 months of age^1^

| Outcomes at 12 months | Predictors at 6 months |  | β (95% CI) |
| --- | --- | --- | --- |
| Infant Z-scored wt gain velocity | Adaptive behavior | Model 1 | -0.04(-0.15,0.07) |
|  |  | Model 2 | -0.03(-0.12,0.06) |
|  |  | Model 3 | -0.02(-0.08,0.04) |
|  | Gross motor | Model 1 | -0.48(-0.60,-0.36)* |
|  |  | Model 2 | -0.35(-0.41,-0.29)* |
|  |  | Model 3 | -0.10(-0.16,-0.04)* |
|  | Fine motor | Model 1 | -0.04(-0.12,0.04) |
|  |  | Model 2 | -0.01(-0.08,0.06) |
|  |  | Model 3 | -0.01(-0.07,0.06) |
|  | Language | Model 1 | -0.03(-0.13,0.07) |
|  |  | Model 2 | -0.02(-0.09,0.05) |
|  |  | Model 3 | -0.02(-0.07,0.03) |
|  | Social behavior | Model 1 | -0.28(-0.32,-0.24)* |
|  |  | Model 2 | -0.20(-0.27,-0.13)* |
|  |  | Model 3 | -0.09(-0.12,-0.07)* |

^1^ N=449. N varied from 1.7% to 2.8% in each regression because the complete data for each subscale of the Gesell Development Scale were varied.

Model 1: adjusted for basic information; Model 2: model 1+ maternal pre-pregnancy BMI, gestational weight gain, delivery mode, gestational weeks, birth weight z score, mode of infant feeding, introduction of solid foods and micronutrients supplementation; Model 3: model 2+ Infant Z-scored wt gain velocity at 6 months in the neurodevelopment- weight gain velocity relationships, or neurodevelopment scores at 6 months in the weight gain velocity – neurodevelopment relationships.

wt: weight;*Statistically significant.

**Bidirectional association of** **neurodevelopment with growth: A Prospective Cohort Study**

**European Journal of Pediatrics**

**Xiaotong Wei^1^, Jiajin Hu^1^, Liu Yang ^2^, Ming Gao^1^, Lin Li^3^, Ning Ding^4^, Yanan Ma^5^ and Deliang Wen^1,^***

* **Correspondence:**

Deliang Wen;

Institute of Health Sciences ,China Medical University, No.77 Puhe Road, Shenyang North New Area, Shenyang, Liaoning Province, 110122, P.R. China (email: dlwen@cmu.edu.cn), (phone: +86 024-31939003).
