# Supplementary material for: Bidirectional association of neurodevelopment with growth: a prospective cohort study
Source: BMC Pediatr. 2021 Apr 28;21:203. doi: 10.1186/s12887-021-02655-7 (PMC8080371; doi:10.1186/s12887-021-02655-7)
Supplement: Supplementary file 5 — Additional file 5: Supplemental Table 5. Longitudinal associations between WFL z-scores and neurodevelopment at 6 and 12 months of age1 [file 12887_2021_2655_MOESM5_ESM.docx]

**Supplemental Table 5** Longitudinal associations between WFL z-scores and neurodevelopment at 6 and 12 months of age^1^

| Outcomes at 12 months | Predictors at 6 months |  | β (95% CI) |
| --- | --- | --- | --- |
| Adaptive behavior | Infant Z-scored WFL | Model 1 | -0.02(-0.04,0.02) |
|  |  | Model 2 | -0.01(-0.03,0.01) |
|  |  | Model 3 | -0.01(-0.04,0.03) |
| Gross motor |  | Model 1 | -0.35(-0.39,-0.30)* |
|  |  | Model 2 | -0.25(-0.32,-0.19)* |
|  |  | Model 3 | -0.10(-0.15,-0.05)* |
| Fine motor |  | Model 1 | -0.02(-0.07,0.04) |
|  |  | Model 2 | -0.01(-0.04,0.03) |
|  |  | Model 3 | -0.01(-0.04,0.02) |
| Language |  | Model 1 | -0.01(-0.05,0.03) |
|  |  | Model 2 | -0.01(-0.05,0.04) |
|  |  | Model 3 | -0.01(-0.03,0.02) |
| Social behavior |  | Model 1 | -0.08(-0.10,-0.06)* |
|  |  | Model 2 | -0.06(-0.12,0.01) |
|  |  | Model 3 | -0.03(-0.07,0.02) |

^1^ N=449. N varied from 1.6% to 2.9% in each regression because the complete data for each subscale of the Gesell Development Scale were varied.

Model 1: adjusted for basic information; Model 2: model 1+ maternal pre-pregnancy BMI, gestational weight gain, delivery mode, gestational weeks, birth weight z score, mode of infant feeding, introduction of solid foods and micronutrients supplementation; Model 3: model 2+ Infant Z-scored WFL at 6 months in the neurodevelopment- WFL relationships, or neurodevelopment scores at 6 months in the WFL – neurodevelopment relationships.

WFL: weight-for-length.

*Statistically significant.

**Bidirectional association of** **neurodevelopment with growth: A Prospective Cohort Study**

**European Journal of Pediatrics**

**Xiaotong Wei^1^, Jiajin Hu^1^, Liu Yang ^2^, Ming Gao^1^, Lin Li^3^, Ning Ding^4^, Yanan Ma^5^ and Deliang Wen^1,^***

* **Correspondence:**  Deliang Wen;

Institute of Health Sciences ,China Medical University, No.77 Puhe Road, Shenyang North New Area, Shenyang, Liaoning Province, 110122, P.R. China (email: dlwen@cmu.edu.cn), (phone: +86 024-31939003).
